# Surveying Europe’s Only Cave-Dwelling Chordate Species (*Proteus anguinus*) Using Environmental DNA

**DOI:** 10.1371/journal.pone.0170945

**Published:** 2017-01-27

**Authors:** Judit Vörös, Orsolya Márton, Benedikt R. Schmidt, Júlia Tünde Gál, Dušan Jelić

**Affiliations:** 1 Department of Zoology, Hungarian Natural History Museum, Budapest, Hungary; 2 Molecular Taxonomy Laboratory, Hungarian Natural History Museum, Budapest, Hungary; 3 Institute for Soil Sciences and Agricultural Chemistry, Centre for Agricultural Research, Hungarian Academy of Sciences, Budapest, Hungary; 4 Department of Evolutionary Biology and Environment Studies, University of Zurich, Zurich, Switzerland; 5 Koordinationsstelle für Amphibien- und Reptilienschutz in der Schweiz, Neuchâtel, Switzerland; 6 Croatian Institute for Biodiversity, Zagreb, Croatia; National Zoological Park, UNITED STATES

## Abstract

In surveillance of subterranean fauna, especially in the case of rare or elusive aquatic species, traditional techniques used for epigean species are often not feasible. We developed a non-invasive survey method based on environmental DNA (eDNA) to detect the presence of the red-listed cave-dwelling amphibian, *Proteus anguinus*, in the caves of the Dinaric Karst. We tested the method in fifteen caves in Croatia, from which the species was previously recorded or expected to occur. We successfully confirmed the presence of *P*. *anguinus* from ten caves and detected the species for the first time in five others. Using a hierarchical occupancy model we compared the availability and detection probability of eDNA of two water sampling methods, filtration and precipitation. The statistical analysis showed that both availability and detection probability depended on the method and estimates for both probabilities were higher using filter samples than for precipitation samples. Combining reliable field and laboratory methods with robust statistical modeling will give the best estimates of species occurrence.

## Introduction

Subterranean ecosystems are among the biomes with the highest number of narrowly distributed and relict taxa [1–3]. This is related to the geographic isolation of subterranean habitats, which facilitate evolutionary drift [4,5]. It is also explained by the lack of Pleistocene glaciations, as these well-buffered habitats were not affected by climatic fluctuations for long periods of time [2,4,6]. Traditionally, compared to terrestrial biomes, subterranean habitats were considered to be less species rich [1]. However, based on the findings of the last few decades and the recently described high incidence of cryptic diversity mostly in invertebrates [4,7–9], this opinion should be revised. While the obligate subterranean fauna is dominated by invertebrates [4,10,11], bony fishes and salamanders were able to successfully colonize these habitats [1,12–14].

In comparison to taxa living on or near the surface of the ground, subterranean biodiversity is significantly less studied. Only a small proportion of cave biodiversity has been explored so far, mostly due to physical inaccessibility or inadequate sampling strategies [15]. Besides hosting high levels of endemism, groundwater biodiversity may sustain valuable ecosystem services (e.g. water purification, bioremediation, water infiltration and transport), therefore it is important to assess population, species and ecosystem diversity of subterranean habitats [16,17].

For surveillance of subterranean fauna, especially in the case of rare or elusive species, effective survey methods are essential. As in underground habitats traditional survey techniques are often not feasible, more sensitive and less invasive tools are necessary [18]. Environmental DNA based detection is currently widely used in aquatic environments, although its advantages in vertebrate species distribution assessments were recognized less than a decade ago [19]. Since then, the application of eDNA has become popular [20], especially since its utility coupled with high throughput sequencing methodologies [21–23]. Due to the high sensitivity and specificity of eDNA it is particularly beneficial for detection of amphibian species which are either rare or hard to spot outside of the breeding season [19,24–27]. The eDNA method could be even more beneficial to subterranean research, by overcoming the physical difficulties of surveying fauna occupying habitats that are inaccessible to humans.

The olm, *Proteus anguinus* Laurenti 1768, is the first ever described cave species, and the only European troglobiont chordate species. It inhabits the underground waters of the Dinaric Karst in the Balkan Peninsula of southeastern Europe, ranging from Trieste in Italy, Slovenia, Croatia to Bosnia and Herzegovina [28,29]. Recent records indicate its presence also in Montenegro [30]. It has been introduced to a cave system at the subterranean laboratory of the French National Center for Scientific Research (CNRS) in the French Pyrenees and to a pit in the German Hartz province [31]. The Dinaric cave area inhabited by *P*. *anguinus* is one of the richest region of underground biodiversity in the world [32].

*Proteus anguinus* has long attracted the attention of researchers due to its troglomorphic characteristics [33,34], longevity [35], ecology [36] and behavior [37,38]. It is listed vulnerable on the IUCN red list [39]. The justification is”Listed as Vulnerable because its Area of Occupancy is less than 2,000 km^2^, its distribution is severely fragmented, and there is continuing decline in the extent and quality of its habitat, and presumably also in the number of mature individuals”. Furthermore, it is recognized as 19^th^ on the list of the EDGE of Existence programme (a global conservation initiative led by the Zoological Society of London to identify the world’s most Evolutionary Distinct and Globally Endangered species (http://www.edgeofexistence.org/)) [40] and is protected by law in Italy, Slovenia and Croatia. Based on literature data and traditional survey methods, such as observations based on visual encounter surveys during cave visits, diving or specimens flushed out by the flow, in Croatia this emblematic species is known from 76 caves [29,41]. As this is only a small fraction of the approximately 7000 caves found in the country [42]—of which most are inaccessible to humans—*P*. *anguinus* is possibly to be much more widespread than hitherto known. The aim of our study was to develop an efficient eDNA-based methodology for the detection of the olm from cave water and to sample several known or putative *P*. *anguinus* locations in the Dinaric Karst in Croatia in order to confirm the efficacy of the method for further application in the conservation of the species.

## Material and Methods

Tissue sampling and research on the olms were approved by the Ministry of Environment and Nature protection of Croatia (UP/I-612-07/11-33/0075, 532-08-01-01-01/1-11-02; UP/I-612-07/15-48/119, 517-07-1-1-1-15-04). Krka National Park provided permission for field work.

### Marker development

We designed a set of species-specific primers for a 60–80 base-pair fragment of the mitochondrial control region using all available sequences of *P*. *anguinus* in NCBI Genbank (DQ494754.1- DQ494786.1) from individuals covering the entire known range of the species. To reduce the chance of cross-amplification with co-occurring amphibian species, when selecting primer binding sites, we compared control region sequences of *Salamandra salamandra* (EU880331.1), *Bombina variegata* (AY971143.1) and *Bufo bufo* (EU627147.1). Sequences were compiled using BioEdit version 7.0.9.0 [43] and aligned manually. During the selection procedure the specificity of the candidate primer pair was assessed *in silico* using the ecoPCR software [44] on the EMBL-Bank release 117 with the following analysis criteria: i) only three mismatches were allowed between the primers and the target sequences, ii) the number of nucleotides with a perfect match on the 3’ end of the primers was two, and finally iii) the minimum and maximum length of the amplicons were 50 and 1500 base-pairs, respectively. The most appropriate primer pair, the so-called “mini-barcode” (Paf8 5’-GTGGCATATAAATCTATGTC-3’ and Par8 5’-TRTTATTCGTTTTCTAGAG-3’) which amplifies a 64 base-pair long fragment, was then further tested in several steps. To calculate physicochemical parameters of the selected oligonucleotides we used the software OligoCalc [45]. The specificity of the 64 bp long target *Proteus* sequence was evaluated using NCBI-Blast [46] against the GenBank Database [47].

### In vitro test of the primers

DNA from *Proteus* tissue samples originating from Miljacka cave, Croatia, were extracted using QIAamp DNA Micro kit (Qiagen, Hilden, Germany) following the manufacturer’s protocol. We included the following non-target species samples: two fish species that are frequently present in cave habitats in Croatia (*Phoxinus lumaireul* and *Squalius illyricus*), and three amphibian species, *Bufo bufo*, *Bombina variegata*, and *Salamandra salamandra*, which are the most common in the area where *Proteus* occurs in the caves, and therefore have the highest chance of „contaminating” the source water.

PCR amplification was carried out in a 10 μl reaction volume containing 1x Qiagen Multiplex PCR Master Mix (Qiagen, Hilden, Germany), 0.5x Q-Solution, 0.2 μM of each primer and 2 μl of template. Touchdown PCR thermal cycling conditions were as follows: 95°C, 15 minutes followed by 15 cycles of 94°C, 30 sec; annealing temperature stepdowns every cycle of 0.5°C (from 60°C to 53°C), 1.5 minutes; 72°C, 1 minute. The annealing temperature for the final 35 cycles was 53°C ending with a 60°C final extension step for 30 minutes. All the PCR reactions were run on a Techne PrimeG thermal cycler (Cole-Palmer Ltd., Vernon Hills, USA). PCR products were run through a 2.5% agarose gel, stained with ethidium bromide and visualized on a UV light platform.

### In situ validation of the detection method

To test the eDNA approach, we used water samples originating from aquarium tanks in Zagreb Zoo (Zagreb, Croatia), where *Proteus* was kept. One individual was kept in about 60 liters of water in an aquarium with the dimensions of 80 x 50 x 30 cm. A field sample was also included, collected from Miljacka cave, Croatia, where a well-known population of *Proteus* exists. 2 L of water samples were filtered with HydroTech Vacuum Pump (Biorad, Hercules, USA) through a sterile 0.45 μm cellulose nitrate filter paper (Kipszer Paraplan, Budapest, Hungary). The filter was cut into small pieces and dried under a sterilized hood.

Extraction was carried out as above with slight modifications of the manufacturer’s protocol, adding double the amount of ATL buffer, proteinase K, AL buffer and ethanol and using QIAshredder columns (Qiagen, Hilden, Germany) after the Proteinase K digestion step.

To enhance the sensitivity of detection, we applied the fluorescent labeling strategy on the forward primer (FAM) used by Goldberg et al. [25]. After amplification, 1 μl of PCR product was mixed with GS500 size standard and Hi-Di Formamide (Applied Biosystems, Foster City, USA) and was run on an ABI 3130 Genetic Analyzer (Applied Biosystems, Foster City, USA), expecting an approximately 64 bp fragment size. Using GeneMarker v.1.80 software (Softgenetics, State College, USA) the fragment with 60 bp length was identified. As the length of our target fragment was at the lower limit of the resolution capability of the Sanger method, confirmation of the amplified product has not been possible via direct sequencing. To overcome this limitation, the fragment was cloned into the pGEM-T Easy plasmid vector (Promega, Madison, USA) and sequenced on an ABI 3130 Genetic Analyzer (Applied Biosystems, Foster City, USA).

For each sample, we carried out four PCR replicates. The sensitivity of the detection was also tested on a dilution series of a known amount of *Proteus* DNA (1X (50 ng/μl), 10X (5 ng/μl), 100X (0.5 ng/μl), 1000X (0.05 ng/μl) and 10000X (0.005 ng/μl)).

### Application of the method on field samples

**Fig 1:**
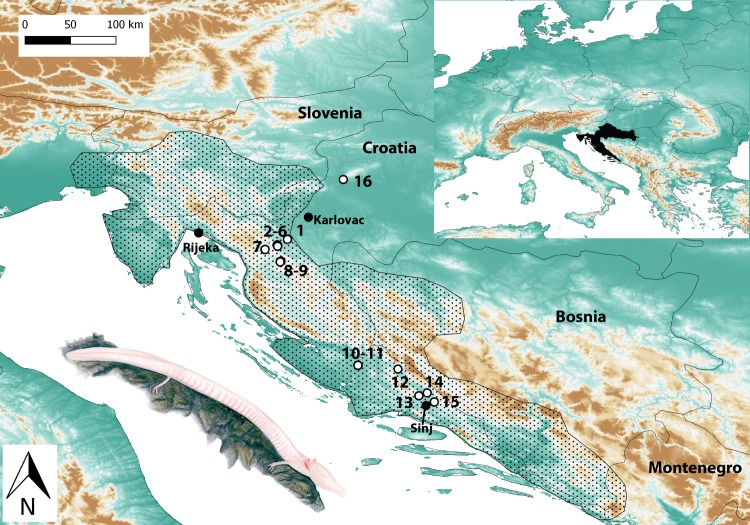
Sampling locations of 16 caves in Croatia. Numbers refer to populations in Table 1. Insert (right) shows location of Croatia in Europe (black). Dotted area shows approximate range of *P*. *anguinus*. Drawing of *P*. *anguinus* is courtesy of Marija Crnčec.

Fresh water samples were collected from 15 localities in Croatia during the summer of 2014, covering most of the regions where the distribution of the species was recently confirmed, data were available in published literature or presence is possible [48–50]. The 15 locations represented several different cave systems (Fig 1, Table 1). Samples were taken either from inside the cave or in case of inaccessibility of the site, from the spring where the water left the cave and reached a natural pond.

**Table 1:** List of sampling locations for detection of eDNA of *Proteus anguinus* in Croatia.

Population	Name of cave or water body	Location and/or city/town/village	Hydro-geological function	Date of last observation	Latitude	Longitude	Precipitation	Filtration
1	Tounjčica	Tounj	Source, cave	This study	45.24	15.32	1/20	1/4
*2	Izvor šiplja Rupećica,	Zagorje,Ogulin	Source, cave	Gottstein Matočec et al., 2002; visual survey in 2014	45.18	15.22	1/20	-
*3	Zeleno jezero (water coming from Ponor Rupećice)	Zagorje,Ogulin	Source, cave	Gottstein Matočec et al., 2002; visual survey in 2014	45.18	15.22	1/20	3/4
*4	Izvor Bistrac	Desmerice, Ogulin	Source, cave	Gottstein Matočec et al., 2002; visual survey in 2014	45.19	15.22	-	1/4
*5	Izvor Zagorske Mrežnice	Desmerice, Ogulin	Source, cave	Gottstein Matočec et al., 2002; visual survey in 2014	45.19	15.22	-	3/4
*6	Jama Klisura	Perakovići, Ogulin	Pit	Jalžić Branko, personal communication, 2012	45.18	15.22	1/20	4/4
7	Zečev studenac	Drežničko field, Drežniča	Source	This study	45.14	15.10	-	1/4
*8	Markarova špilja	Stajnica,Jezerane	Occasional source, pit	Gottstein Matočec et al., 2002; visual survey in 2014	45.03	15.25	14/20	4/4
9	Izvor u Stajničkom polju	Stajničko field, Stajnica	Source	This study	45.02	15.25	-	1/4
*10	Miljacka II	Kistanje, Šibenik	Occasional source, cave	Gottstein Matočec et al., 2002; visual survey in 2014	44.00	16.01	2/20	4/4
*11	Špilja kod mlina na Miljacki	Kistanje,Šibenik	Occasional source, cave	Gottstein Matočec et al., 2002	44.00	16.01	1/20	3/4
12	Vukovića vrelo	Cetina, Civljane	Source, pit	This study	43.96	16.41	-	4/4
*13	Goručica, izvor potoka	Sinj	Source	Gottstein Matočec et al., 2002	43.70	16.61	1/20	4/4
14	Kosinac	Han, Sinj	Source	This study	43.73	16.70	-	4/4
*15	Izvor Grab	Grab, Trilj	Source, cave	Kovač Konrad Petra, personal communication, 2011	43.64	16.77	2/20	3/4
16	Veternica	Medvednica, Zagreb	Cave	Negative control	45.84	15.87	-	-

Localities, hydrogeological function of sampled water bodies, geographic coordinates, number of positive/total samples collected for both precipitation and filtration method for 16 populations of *Proteus anguinus*.

* At locations marked with * presence of *Proteus* is documented in literature or was detected recently by visual survey.

Surveys were done by cave divers, using line transects for monitoring the *Proteus* populations.

Date of publication and/or last visual detection is also given.

During a single visit at each location, five replicates of 15 mL of water closest to the source were collected in 5 x 50 mL Falcon tubes and mixed with the solution composed of 1.5 mL of sodium acetate 3 M and 33 mL absolute ethanol [19]. The samples were then transferred to the lab and stored at -20°C until processing.

To recover the precipitated DNA and/or cell debris, Falcon tubes were centrifuged at 10°C for one hour at 8,000 g. The supernatant was discarded carefully and another centrifugation step was performed on the remaining 5 mL of sample at 10°C for 10 minutes. The supernatant was once more discarded and the remaining one mL of sample was transferred into a tube of 1.5 mL. At this point, a third centrifugation step was introduced at 10°C for 10 minutes to ensure maximum recovery of the eDNA. After discarding most of the supernatant, the pellet was dried at room temperature to evaporate the remaining EtOH. This procedure was followed by the standard DNA extraction method using QIAamp DNA Micro Kit (Qiagen, Hilden, Germany). DNA amplification and fragment analysis were performed following the protocol described in the „*In situ* validation of the detection method” section.

Parallel to sampling for precipitation 2 L of water samples were collected at each location into separate new sterile plastic containers, and were stored on ice until being filtered. Filtration was carried out maximum within one hour from collecting in a nearby accommodation. Each filter paper was preserved in 96% ethanol in separate sterile 2 mL tubes, transferred to the lab and stored at -20°C until processing.

After the filter was cut into small pieces and dried under a sterilized hood, extraction, DNA amplification and fragment analysis were performed following the protocol described in the „*In situ* validation of the detection method” section.

### Quality and negative controls

In order to avoid contamination in the field additional equipment was sterilized between locations using EtOH and flame (scissors and forceps) or Alconox (Sigma-Aldrich, St. Louis, USA) detergent solution (parts of filtering machine) and rinsed with tap water. As a negative location control, we used a fresh water sample taken from Veternica cave (Medvednica Mts.) near Zagreb (sample 16 in Table 1), which is not part of the Dinaric karst system and is far outside of the species distribution range [29,41]. At every extraction event we filtered 2 L of sterile water through a sterile filter paper which was processed parallel with the samples taken from the location. The negative extraction control, negative PCR setup control (using the same set of reagents but instead of template we loaded 1 μl of sterile water), and positive PCR setup control (10 ng/μl *Proteus* DNA extracted from tissue sample) was included in every reaction.

### Statistical methods

We used a hierarchical occupancy model to estimate cave occupancy probabilities and detection probabilities [51]. The model uses multiple water samples and multiple PCR per water sample to decompose detection probability into two components. The first component is the availability probability of eDNA in the water sample and the second component is detection probability in the PCR [51]. Both probabilities contribute to false negative error rates. We used the Bayesian software WinBUGS and the R package “R2WinBUGS” to fit the model to the data. WinBUGS and R code were taken from Schmidt et al. [51] and the analysis carried out as recommended [52] using uniform vague priors [51]. We modeled availability and detection probabilities as a function of the method (precipitation vs. filtration). The dataset we used is available as a supplementary material in S1 Table.

## Results

Even though our target sequence of 64 bp was shorter than the recommended 90–120 bp length [53], its specificity was confirmed using various tests described below. The *in silico* analysis indicated the specificity of the primers and resulted in no co-amplifying species at three mismatches between the primers and the target sequences. The *in vitro* specificity of the primers was confirmed as they did not amplify any of the tested co-occuring species (*Salamandra salamandra*, *Bufo bufo*, *Bombina variegata*, *Phoxinus lumaireul* and *Squalius illyricus*). The NCBI Blast search resulted in 34 hits with 100–98% identity to *Proteus anguinus* D-loop sequences.

**Fig 2:**
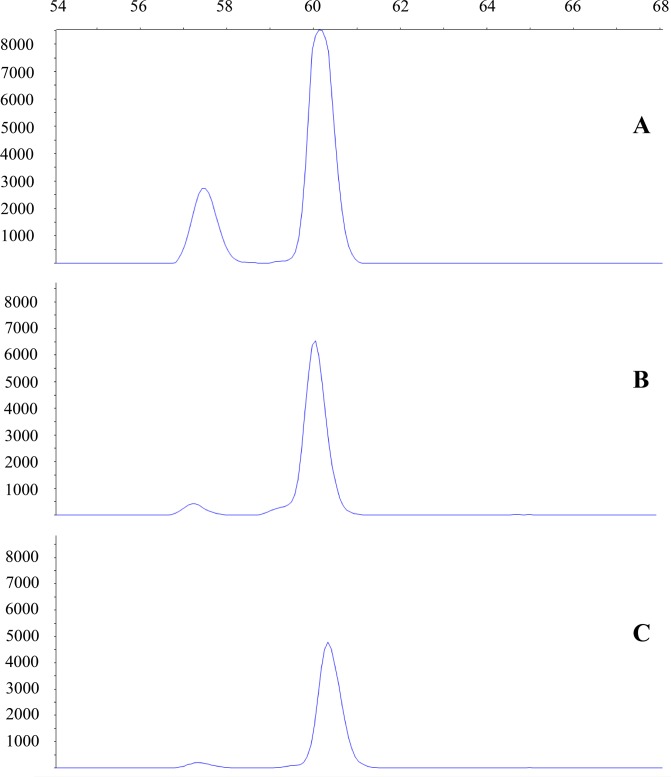
60 bp long fragments produced with fluorescent labeled primers. The blue peak indicates the species specific fragment for *Proteus anguinus* in positive control from tissue sample (A), in a sample collected with filtering 2 L of water (B) and in a sample collected in the solution composed of 1.5 mL of sodium acetate 3 M and 33 mL absolute ethanol (C).

PCR with fluorescently labeled primers provided clear detection of amplified fragments (Fig 2). During the validation via sequencing the 64 bp fragment was successfully cloned for environmental samples and the specific *Proteus* fragment was recognizable from the plasmid sequence (S1 Fig). During the cloning procedure neither primer dimer nor non-specific sequences were detected.

The correct fragment was detectable down to 10000X (0.005 ng/μl) DNA concentrations. When applying the method on field samples we successfully amplified DNA from water samples from nine locations using the precipitation method and from fourteen locations using the filtration method (Table 1). We never amplified *Proteus* DNA from the negative control location, negative extraction or negative PCR controls. We successfully amplified DNA of *P*. *anguinus* from every positive PCR setup control.

The statistical analysis using the hierarchical occupancy model [51] showed that both availability and detection probability depended on the method (Fig 2). The posterior distributions overlapped very little. Availability was 3.78 times higher in filter samples than in tube samples and detectability in PCR was 1.90 times higher for filter samples than for tube samples. Based on the model, the estimated number of occupied caves is 15 (95% credible interval: 15–15; based on finite sample estimation [54]).

## Discussion

We successfully developed a non-invasive detection method for the endangered and elusive amphibian species, *Proteus anguinus*, using environmental DNA. Although DNA metabarcoding is more useful and cost-efficient when detecting several target organisms at the same time [55], because of the need of high specificity and sensitivity to identify *P*. *anguinus* DNA from cave water, we opted for a single-species and single-marker detection approach. Previous studies showed that environmental conditions, biomass and production rate of specimens strongly influence detectability of organisms [27,51,56]. Barnes et al [57] reviewed the environmental factors that affect eDNA persistence and showed that abiotic factors, e.g. temperature, ultraviolet radiation and light exposure has negative impact on DNA degradation. Effects of abiotic and biotic factors on detectability in these habitats are unknown because we could not do experiments using our target species and very few studies have investigated eDNA dynamics in caves [58]. Nevertheless, caves inhabited by *P*. *anguinus* represent an environment with climate buffered against weather fluctuations, darkness and cold water all year round which may help eDNA to persist for longer than in surface waters.

Klymus et al [56] showed that in the case of fish (bighead and silver carp) higher rates of water flow may hamper detectability of eDNA from flowing water systems. Therefore it is important to carefully choose the most appropriate time for sampling events. For *P*. *anguinus*, prior knowledge of the water levels of the actual cave system is necessary to perform eDNA sampling. Preliminary studies have shown that high water level due to natural conditions such as snow melting or heavy rain mostly in springtime resulting in fast waterflow of underground water systems, can decrease the chance of eDNA detection [59]. Additionally *P*. *anguinus* is considered a low energy vertebrate, with the ability to withstand long-term starvation presumably due to the sporadic food supplies seen in hypogean environments [60]. *Proteus anguinus* may also have a lower shedding rate compared to surface-dwelling vertebrates. This is significant because vertebrate shed cells are an important source of eDNA [53] and some studies of fish have shown that they shed faster in environments with higher food intake [56]. Since cave water is characterized by limited food resources, it might contain fewer vertebrate shed cells than epigean environments. Unfortunately, there are no studies of the correlation between food intake and shedding rate of *P*. *anguinus* so, targeted experiments are needed to explore whether these factors influence eDNA detectability.

**Fig 3:**
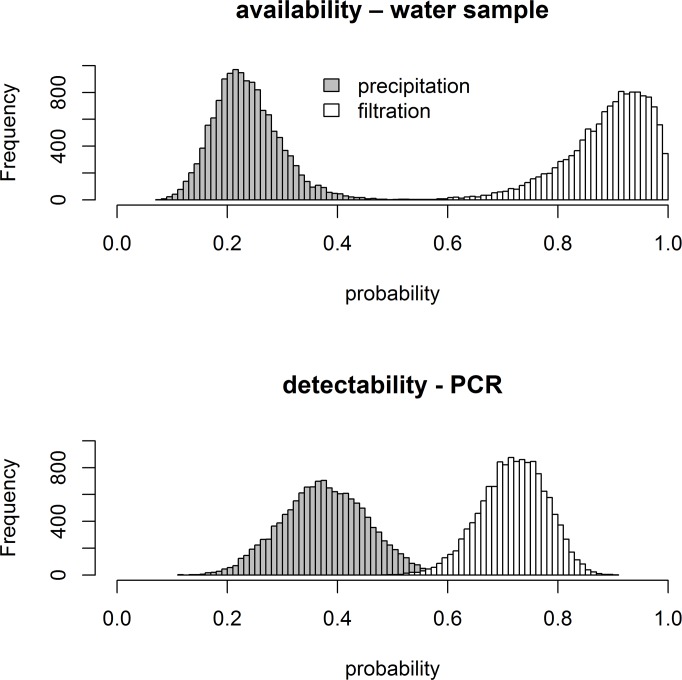
Posterior distributions of model parameters. The figure shows the posterior distributions for availability and detectability probabilities for the two methods, filtration and precipitation.

When designing an eDNA monitoring approach other important factors, such as the choice of eDNA capture method or the level of replication (i.e. number of water samples, number of PCR) have to be considered. Here, we tested the efficiency of filtration and precipitation methods under restricted sampling conditions. Our study showed that despite the much higher PCR replication level, the precipitation method failed to detect the species in six localities, where the filtration method gave reliable signal. Thus, there are false negatives, which imply that detection is imperfect, which is reported by most studies [51,61]. We therefore used a hierarchical site occupancy model to account for detection error [51]. The results of the site occupancy analysis quantified false negative rates (i.e. availability and detection probabilities) and statistically confirmed that both availability and detection probability of *Proteus* eDNA was higher using the filtration method than the precipitation method in cave environments. Our results also suggest that detection error is about equally likely to occur at the water collecting stage as it is at the PCR stage (Fig 3). Even though error rates are low when the filtration method is used, replication at both stages seems worthwhile and necessary. These findings are concordant with several other studies confirming that in flowing water bodies more eDNA can be recovered using filtering of large volume of water, while the precipitation method can be useful for studying species in stagnant waters [18,24,25], where the collection of more subsamples is not restricted. The performance of filtration is strongly influenced by the availability of the eDNA in the environment (the ratio of intra- and extracellular DNA) and the increased presence of possible inhibitors collected by larger amount of water [62]. The amount of eDNA present in a water body can be influenced by the density of species [19,24,26], but as population density data is lacking for *Proteus* (and most other subterranean animals) we were unable to test this factor. In conclusion, we believe that one should work as carefully as possible both in the field and in the laboratory to minimize detection errors. However, experience shows that there is almost always some level of imperfect detection in surveys in general and in eDNA survey studies in particular [51,61]. We therefore recommend the application of hierarchical models to eDNA data because the combination of state-of-the-art laboratory, field and statistical methods should yield the most reliable estimates of the number of occupied sites and prevent the under- or overestimation of those quantities of interest [51,63]. We note that occupancy models can also be used to estimate false positive error rates [63].

In practical terms, the main differences between the two methods (precipitation and filtration) are the particle fraction of the eDNA pool they are targeting and the starting sample volume, consequently the total amount of DNA they are operating with. The precipitation method is able to collect even the extracellular DNA fractions (usually < 0.2 μm), but from a small water volume (15 mL). In contrast, the filtration method can capture the eDNA from a hundred-times larger starting sample volume (2 L), but with a focus on a narrower, intracellular particle size spectrum depending on the filter pore diameter. To minimize the chance of false negative events (the species is present in the environment, but the monitoring method is unable to detect it) in rare, endangered and/or invasive species monitoring is one of the main challenges in eDNA studies. Deiner et al. [64] suggested in their comparative study that the combination of the filtration technique (e.g. applying sequential filtration) and the proper DNA extraction kit can effectively reduce this issue.

With our newly developed eDNA detection method we confirmed the presence of *P*. *anguinus* from ten caves in Croatia, and detected the species for the first time in five others. All new localities are located within the generally known range of *P*. *anguinus* but they do add new data on the distribution of specific biogeographic areas. Locality 1 (Tounčica), locality 7 (Zečev studenac) and locality 9 (Izvor u Stajničkom polju) are part of Gorski kotar (a mountanous region between Karlovac and Rijeka, Fig 1) population, concentrated around the Zagorska Mrežnica River basin. Here, the presence of *P*. *anguinus* has been known for a long time, thus new data on distribution were expected. Locality 12 (Vukovića vrelo) is one of the two main springs of Cetina River and it has been dived to the depth of 105 m but no *Proteus* has been detected until now. Locality 14 (Kosinac) is a spring of the small left tributary of the same river (Cetina). Divers searched for *P*. *anguinus* but observed none. In both caves of localities 12 and 14 *Salmo farioides* occurs and the presence of this predator could explain why no *P*. *anguinus* have been observed before. They might retreat into deeper parts of the cave system which are inaccessible to large fish and humans. From locality 13 (Goručica) *Proteus* is only known from historical data and was not reconfirmed during recent field studies. The source (spring) of Goručica was heavily affected by human activities in the past as it was one of the main water sources for the city, Sinj. This left the source filled with large rocks and completely inaccessible. However, our results indicate that there is still a population within the underground system. This subpopulation—including localities 12, 13 and 14—belongs to the isolated population of *P*. *anguinus* classified as Cetina River population.

To assess conservation status and to establish reliable conservation plans on rare or threatened species accurate distribution data are key elements [65,66]. Reliable spatial data offer opportunities e.g. for species distribution modeling and to assess the impacts of climate change on species. In this study, we provided a tool that can help to locate new caves where the elusive *P*. *anguinus* lives, obtaining data valuable for conservation planning. Further surveys are vital for producing good quality data on distribution and help to provide a basis for decision-making in conservation.

## Supporting Information

S1 Fig Sequences of cloned DNA fragments inserted into the plasmid sequence. (TIF)

S1 Table Table including locality details and data analysed in this study. (XLSX)
