# Supplementary figures and images for: Surveying Europe’s Only Cave-Dwelling Chordate Species (Proteus anguinus) Using Environmental DNA
Source: PLoS One. 2017 Jan 27;12(1):e0170945. doi: 10.1371/journal.pone.0170945 (PMC5271363; doi:10.1371/journal.pone.0170945)

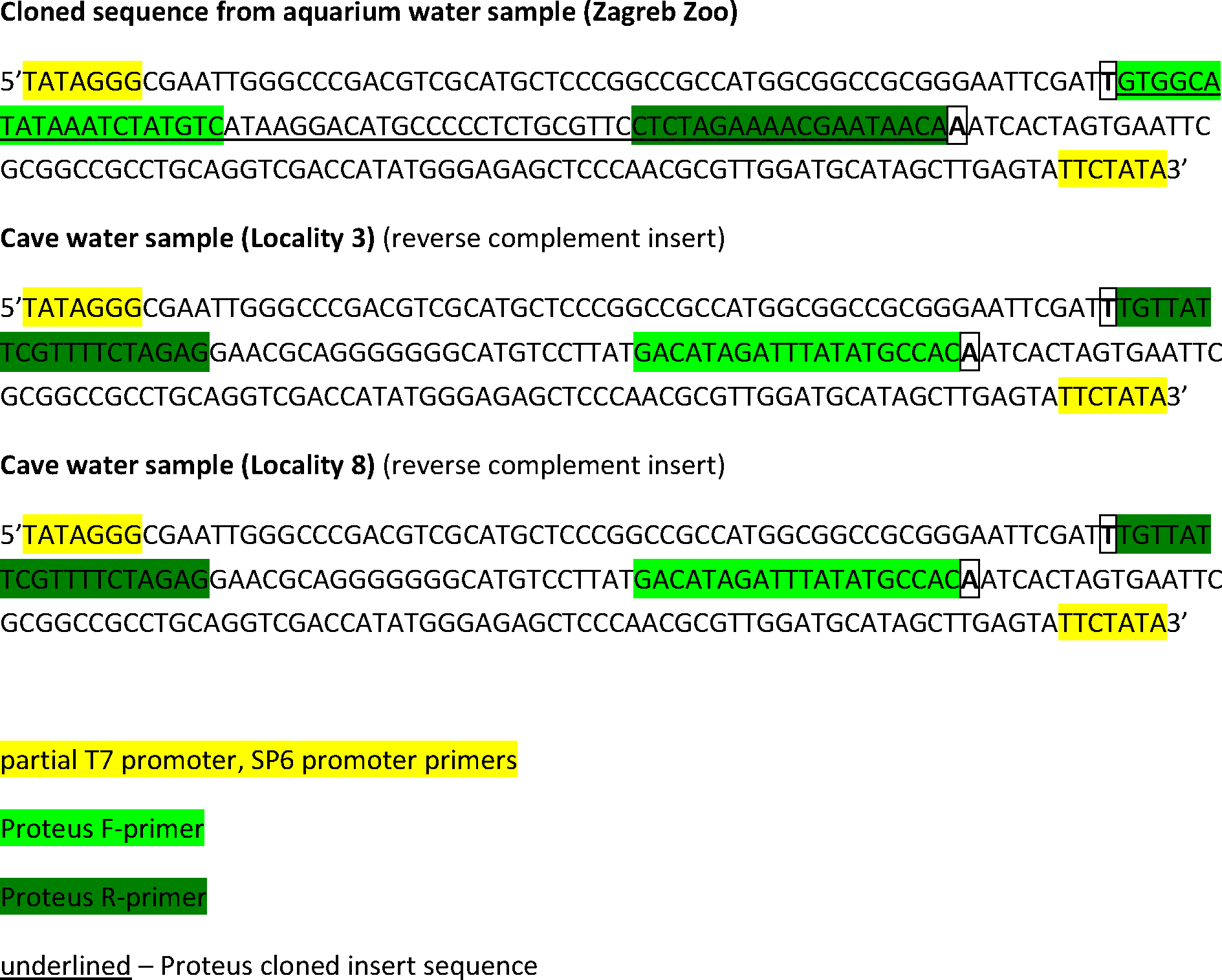

Supplement: S1 Fig — (TIF) [file pone.0170945.s001.tif]
